# The role of human rights in implementing socially responsible seafood

**DOI:** 10.1371/journal.pone.0210241

**Published:** 2019-01-25

**Authors:** Lydia C. L. Teh, Richard Caddell, Edward H. Allison, Elena M. Finkbeiner, John N. Kittinger, Katrina Nakamura, Yoshitaka Ota

**Affiliations:** 1 The Nippon Foundation Nereus Program, Vancouver, Canada; 2 Institute for the Oceans and Fisheries, University of British Columbia, Vancouver, Canada; 3 School of Law and Politics, Cardiff University, Cardiff, United Kingdom; 4 School of Marine and Environmental Affairs, University of Washington, Seattle, WA, United States of America; 5 CGIAR Research Program on Fish, WorldFish, Penang, Malaysia; 6 Center for Ocean Solutions, Stanford University, Monterey, CA, United States of America; 7 Conservation International, Center for Oceans, Honolulu, HI, United States of America; 8 Arizona State University, Center for Biodiversity Outcomes, Julie Ann Wrigley Global Institute of Sustainability, Life Sciences Center, Tempe, AZ, United States of America; 9 Conservation International, Betty and Gordon Moore Center for Science, Arlington, VA, United States of America; 10 The Sustainability Incubator, Honolulu, HI, United States of America; Department of Agriculture and Water Resources, AUSTRALIA

## Abstract

Sustainability standards for seafood mainly address environmental performance criteria and are less concerned with the welfare of fisheries workers who produce the seafood. Yet human rights violations such as slavery and human trafficking are widespread in fisheries around the world, and underscore the need for certification bodies and other seafood supply chain actors to improve social performance, in addition to addressing environmental challenges. Calls for socially responsible seafood have referenced human rights law and policy frameworks to shape the guiding principles of socially responsible seafood and to provide the legal machinery to implement these aspirations, but practical guidance on how to achieve this is lacking. To provide clarity on this challenge, we reviewed the literature concerning human rights in the seafood supply chain, and prepared an analysis of opportunities and challenges to implement socially responsible seafood through relevant human rights, legal and policy instruments. We observe that human rights laws are generally framed in favour of addressing violations of civil and political rights, but there remains considerable scope for applying economic, social and cultural (ESC) rights in this context. Other challenges include weakly defined ESC rights infringements, a lack of straightforward mechanisms to enforce human rights entitlements, and practical difficulties such as resources to support and secure rights. On the positive side, governments can draw on international instruments to inspire national policies and legislation to eliminate illegalities from the seafood supply chain. However, for socially responsible seafood principles to translate into tangible actions, these objectives must be rooted in clear legal obligations and be supported by sufficient national capacity and political will.

## Introduction

The sustainability movement in the seafood sector has grown significantly over the past twenty years in response to investments in market-based incentives and governance improvements. In 2015, seafood that was certified as environmentally sustainable constituted 14% of global seafood production, compared to just 0.5% in 2005 [1]. The demand for seafood certified as sustainable reflects concerns over declining fish stocks and a desire to protect the oceans from becoming overfished. A number of ratings, certifications, and ecolabels have emerged to assure consumers of particular aspects of sustainability [2]. However, it has become increasingly clear that environmental sustainability is not the only challenge facing seafood production. Recent exposures of exploitative labour practices in the seafood supply chain, including slavery and human trafficking, clearly demonstrate a systemic disregard for human well-being within some sectors of the fishing industry [3,4].

While slavery and slavery-like practices represent one especially egregious example of the violation of people’s civil and political (CP) rights during the production of seafood, they are but one of a pervasive set of social injustices experienced by fisheries workers [5]. More latent and widespread violations include actions that perpetuate discrimination, deny fair access and sharing of benefits, and threaten food and livelihood security in fishing communities [6]. Such actions are largely infringements of people’s economic, social, and cultural (ESC) rights, which aim to ensure that individuals have the freedoms and protections required to live a dignified life, such as the right to decent work, education, health care, and cultural identity. Actions that deny people their ESC rights contribute to increased vulnerability and insecurity in people’s lives, which further impedes marine stewardship for long-term resource sustainability [7–9]. Therefore, continued violations of those rights supporting human dignity in seafood production undermine the socio-economic sustainability of fisheries and ought to be addressed with the same concentrated efforts accorded to other threats to rational management, such as overfishing and ecologically unsustainable practices [7]. However, to date seafood certifications have predominantly focused on promoting environmental sustainability while largely ignoring socio-economic considerations [10,11].

Heightened awareness by the public and law-makers of social abuses in the global seafood industry is increasingly requiring companies to find solutions to eliminate violations in their supply chain, hence this is now an opportune time to reframe sustainable seafood around an ethical core which industry can then endorse and use to build social responsibility into seafood production [12,13]. Advancing a more central recognition of human well-being in seafood production can help to comply with national laws and international obligations concerning human and labour rights, as well as more aspirational commitments such as the poverty alleviation and food security targets of the United Nations (UN) Sustainable Development Goals [14]. At the same time, many of the commitments enshrined within the legal and political framework for the protection of human rights are also pertinent to the pursuit of socially responsible seafood.

Demand is rising for the promotion of socially responsible seafood more centrally within the marketplace [12]. The social discourse has relied on the overarching framework provided by human rights laws to shape guiding principles and to provide the necessary legal machinery to protect and enforce human rights in seafood work. This demand is pushing the seafood sustainability movement into new territory. Thus far, seafood sustainability has largely been equated with fisheries that are managed to achieve particular ecological and environmental objectives, as reflected in seafood certifications such as the Marine Stewardship Council (MSC) ecolabel, and ‘dolphin-safe’ tuna. Social responsibility is an entirely different paradigm, and one which brings stakeholders accustomed to ecological issues into the less familiar realm of human rights and social development. A steep learning curve therefore lies ahead for seafood sector stakeholders to reconcile these two disparate fields, and to effectively navigate the nexus between sustainable seafood and human rights.

This paper explores the extent to which a recognised social responsibility standard is reinforced by existing human rights legal and policy instruments. Our analysis shows that the international, regional and domestic frameworks for the protection of human rights could be more directly harnessed to combat social abuses and other harmful practices in the seafood supply chain that contradict the principles of socially responsible seafood. However, we also counsel that a degree of caution is appropriate in gauging the alignment of business and human rights approaches in this respect, since the legal regimes established for the protection of human rights are not expressly designed to promote socially responsible practices as a central objective. Nevertheless, the objectives of socially responsible seafood and of particular human rights instruments are not necessarily mutually exclusive. In this paper we i) review the definition of ‘socially responsible seafood’ within the context of human rights frameworks; ii) assess the scope of social concerns in the seafood supply chain; and iii) examine the opportunities and challenges to implementing socially responsible seafood through the broad framework of human rights, considering the most pertinent legal and policy instruments that may be applicable to the context of socially responsible seafood. Finally, we draw upon these qualitative analyses to identify future avenues through which to incentivise the production of socially responsible seafood and to discourage exploitative and discriminatory practices.

### Finding space for social safeguards in sustainable seafood

Existing seafood certifications, ratings, standards, and assurance programmes are mainly concerned with environmental sustainability and, with a few notable exceptions (e.g., Fair Trade Capture Fisheries Standard, Seafish Responsible Fishing Scheme) [15,16], the social dimensions of sustainability have been noticeably absent from this space. For example, human rights are absent from the MSC standard, and labour practices were only recently considered as a self-reporting requirement for applicants to MSC’s fishery and chain-of-custody programmes [17]. For environmental issues, certification and ratings programmes have been effective market-based initiatives in driving commitment among businesses to shift their procurement and supply chain management practices towards sustainability [18]. Today, around 90% of the North American grocery retail market has made sustainable seafood commitments [19], and industry and non-profit groups are currently focused on implementing these commitments to drive environmental improvements in fisheries and aquaculture performance, primarily through alignment with the current standards. This notwithstanding, certification and ratings schemes do have their own challenges, including documented examples of certified fisheries in stocks that were not improving or even overfished, the lack of a mechanism to prevent certified stocks from being simultaneously fished by harmful methods, and the questionable end use of certified fisheries [20–22].

While environmental improvements are ongoing, an equivalent approach to send a strong market-signal for social responsibility has yet to fully emerge. Some companies are beginning to consider ethical and responsible sourcing [23] but primarily focus on labour issues that do not encompass a broader array of socio-economic and cultural dimensions affecting small-scale producers and developing economies [6]. This gap can potentially undermine the environmental, social, economic and institutional sustainability of fisheries [7]. For instance, the development of new fisheries for certified seafood may lead to unanticipated impacts such as increased pressure on existing fish stocks and decreased local availability of fish protein supply, or widening social inequity in resource benefit distribution [24]. Nonetheless, the experience derived from environmental certifications and ratings indicates that equivalent efforts to incentivize social responsibility could be impactful [25]. To be successful, efforts to secure socially responsible seafood through certification must also be buttressed by effective legal mechanisms and political will at both a national and international level.

Several ongoing efforts are conducive to this endeavour. First, a broad consortium of organizations came together to create a shared definition of social responsibility for the seafood sector, known as the ‘Monterey Framework’, which drew upon the UN Food and Agriculture Organization (FAO) Guidelines for Small-Scale Fisheries (SSF Guidelines) [26] and incorporated a range of relevant literature and practical experience [12]. This definition is incorporated into the Conservation Alliance for Seafood Solutions’ Common Vision for Sustainable Seafood [27] and the Seafood Certification and Ratings Collaboration’s Framework for Social Responsibility [28]. The latter is developing a benchmarking protocol to assess social performance in the sector [29], and applying the Monterey Framework to the protocol in fisheries improvement projects. Second, new technologies are being piloted in the seafood sector to improve transparency and accountability, creating mechanisms for increased worker protection and worker voice. Lastly, some industry organisations, together with support from non-profit and philanthropic groups, have begun dialogues to support the adoption of best practices in the sector.

Even with this progress, the majority of seafood production takes place with little in the way of social safeguards for migrant and subcontracted workers and limited acknowledgment of inherent links between social responsibility and environmental sustainability [12,30]. Labour abuse acts as a perverse subsidy to overfishing and increasing demand for imported seafood continues to drive decreases in social equity concerning resource benefit distribution. Mitigating these negative impacts requires a clear understanding of the inherent socio-ecological characteristics of the producer communities. Underlying socio-economic dynamics and governance systems in developing countries can impede the progress of fisheries improvement projects geared towards MSC certification [31], while fishers who lack certain access rights, knowledge, or capital equipment may inadvertently lose out on trade benefits due to the export orientation of seafood supply chains and trade dynamics [32]. Improving social well-being in seafood production can thus be most fully realised by focusing on the entire supply chain, from contextual complexities in producer communities [33] to the middle of the supply chain, and buyer commitments and their procurement policies.

### The Monterey Framework

This framework defines social responsibility in the seafood sector as comprising three core pillars: (1) Protect human rights and dignity, and respect access to resources, particularly for indigenous and vulnerable populations; (2) Ensure equality and equitable opportunities to benefit from such access; and, (3) Improve food and livelihood security [12]. These criteria necessarily extend across production modes (i.e., are inclusive of both aquaculture and wild-capture fisheries, and small-to-large scale production systems) and along the entirety of the supply chain. The framework recognises a spectrum of human rights from flagrant violations, such as freedom from slavery and forced labour, to more subtle infringements such as systemic discrimination, shortfalls in due process and inequitable outcomes in the allocation of fishing rights, access, and benefits.

## Methods

The definition of socially responsible seafood used in this paper follows the three pillar Monterey Framework introduced in Kittinger et al. [12]. Using this framework, we reviewed social concerns in the seafood supply chain via a desktop literature search to identify and document the range of human rights violations that fall within scope. We started by conducting an internet search of the topic ‘human rights violations in fisheries’ using the Google search engine web browser (See S1 File for search strings). We considered all types of source materials that the search engine returned, which included academic publications, online newspapers and media, and reports, working papers, and other grey literature by governments, international institutions (e.g. FAO, International Labour Organization (ILO)), seafood sustainability non-profit organisations (e.g. Fishwise), front-line human rights organisations (e.g. Human Rights Watch), and non-governmental organizations. The types of human rights violations that this search returned covered human trafficking, forced labour, health and safety violations, child labour, slavery, and multiple other on the job abuses (long working hours, unpaid wages, physical and/or mental abuse, murder at sea). A second refined internet search was conducted for the specific human rights violations that were brought up in the first search.

Many types of human rights infringements in fisheries are not as blatant as the likes of slavery, and are not acknowledged or labelled as such. An initial search for more subtle violations of economic, social and cultural rights in fisheries did not produce additional case examples (see S1 File for search strings). We found that non-labour rights violations were not discussed explicitly as rights violations, but rather in the context of interventions like fisheries management, coastal planning, or marine conservation. Therefore, we focused on identifying the consequences of not securing economic, social and cultural rights in small-scale fisheries as outlined in Sharma [34], which included rights to coastal resources and participation in their management, access rights to fisheries resources and fishing grounds, inclusive fisheries management, traditional knowledge and cultural rights, and rights of fair access to markets, credit and trade. We then conducted specific searches for characteristics of social injustice and poor management in fisheries. Outstanding issues that emerged were unequal distribution of benefits, marginalisation and exclusion of minorities, communities, and traditional knowledge from decision-making, lack of respect for diversity and customary systems, loss or disruption of socio-economic stability and livelihoods, and food insecurity. Finally, we drew on human rights instruments to discuss the ability of the current legal framework to tackle human rights infringements in fisheries.

## Results

### Refining the human rights landscape of socially responsible seafood

Here we explore the scope for potential human rights violations in fisheries within the three pillars of socially responsible seafood encompassed in the Monterey Framework, as informed by the literature search.

### Protect human rights and dignity

This principle calls for respecting basic human rights and dignity and protecting labour rights, as well as securing access to resources. Failure to adhere to this principle can result in violations such as forced evictions, child labour, forced labour, detention without trial, and violence against fishing communities, all of which were evident in a review of human rights abuses in fisheries [6]. Incidences of physical abuse and unsafe working conditions for fishers, corrupt manning agents, victimisation, unpaid wages and unlawful detention were further documented by ITF [35]. Denying people access to resources has wide ranging socio-economic impacts which, in this paper, we will discuss more thoroughly in the sections on equity and equality, and food and livelihood security.

Since 2000, a number of reports have focussed on forced labour and human trafficking in the fishing sector, and are described in ILO [36]. Most recently, a major international news journal documented the plight of migrant workers aboard Thai fishing boats–involving men from neighbouring countries of Cambodia or Myanmar who are trafficked across the border and sold into years of modern day slavery aboard Thai fishing vessels [4,37–39]. One study suggests that the number of migrant workers in Thailand who are forced to work in the fishing sector, either on fishing boats or in fish processing, is significantly higher than that in the agriculture sector [40].

Child labour has been also reported in the fish harvesting and processing industries of developing countries, with estimates suggesting that there may be “many millions” of child labourers involved in fisheries and aquaculture throughout the world [41]. In Ghana, children as young as four are sold as bonded labourers to work in the Lake Volta fishery, where they are exposed to the ‘worst forms of child labour’, defined by the ILO as all forms of slavery, trafficking, and work that is likely to harm the health, safety, or morals of children (ILO Convention No. 182 Art. 3). Here, children are denied basic education, malnourished, and forced to do hazardous tasks including hauling heavy fishing nets and diving in dangerous conditions to untangle nets [42]. Since 2002, over 700 children, some whom have been held in conditions of slavery for 10 years, have been rescued from Lake Volta’s fishery [42]. Nor are industrial workplaces in major seafood hubs immune. For example, there is evidence that children aged 5 to 17 are engaged in fishing and fish processing in Vietnam, of whom all those in fishing and more than 80% of those in fishing processing were involved in work that could be considered hazardous according to national legislation [43]. Human trafficking in the fishing industry also includes that of boys and young males to work on board commercial fishing vessels in Asia and globally [44].

These concerns are not confined to developing countries. Indeed, in 2015, investigative reporters at *The Guardian* raised allegations of trafficking within elements of the Irish trawling industry [45], while in February 2018 the Seafood Slavery Risk Tool (http://www.seafoodslaveryrisk.org/) implicated certain UK scallop fishers as being complicit in human trafficking and bonded labour [46]. Human trafficking in New Zealand’s fishing industry was exposed by a number of researchers [5,47–49]. Vulnerable workers from countries such as Indonesia and the Philippines are coerced and trafficked by agents into New Zealand, where they are allegedly bonded into forced labour aboard a number of foreign chartered vessels. These foreign vessels are legally leased by New Zealand companies to catch fish in New Zealand waters to fulfill nationally allocated catch quotas and as such the crew should be subject to New Zealand labour law standards. Since 2005, more than 10 instances have been documented of crew defecting from Korean and Ukranian owned fishing vessels to escape abusive treatment. On top of the physical and mental trauma they have already suffered, these crew members are often further abused by being denied their promised wages. According to Harré [47], many elements of human trafficking are evident in New Zealand’s fishing industry, including “dealing in slaves; money laundering; dishonesty offences; false accounting practices; deception of government officials, and natural resource crime”.

Finally, labour conditions on fishing vessels can be inherently challenging, even where vessels are well managed, with fishing considered one of the most hazardous occupations based on reported fatality statistics. Fatality rates in fisheries can range from 3.5 times (Canada) to 15 times (Republic of Korea) above the national average [50], and are likely to be much higher in developing countries where data are less available. While fatalities at sea are not always the result of violations of human rights or applicable maritime law, the safety of fisheries workers is nevertheless more frequently compromised in situations where human and labour rights are not respected [5]. For example, migrant workers working on Irish trawlers allegedly earn less than minimum wage, work for very long hours and experience unacceptable levels of workplace accidents [51].

### Ensure equality and equitable opportunities to benefit

This principle is concerned with social justice and fairness in the seafood supply chain. It pertains to issues such as non-discrimination, equitable distribution of benefits to workers and inclusiveness in representation and participation in decision-making. This principle also addresses less conspicuous threats to small-scale fishers that arise from poor governance and oversight on social justice, including potential erosion in access to fish resources that can drive loss of livelihood and human security [34].

The ability of fishers to pursue fishing opportunities is influenced by the resources they possess, the rights afforded to them, and their relative power to mobilise these resources and rights [52,53]. Fishers’ access to resources, be they natural, financial, or human, however, are not equal and, among other factors, is influenced by legal recognition [54], political-economic power, kinship ties, social status, and ecological knowledge [55]. These inequalities exist even within individual communities and tend to get lost when individual fishers are grouped together as a homogenous ‘community’ stakeholder.

Perceived inequality among stakeholders can lead to disputes and mismanagement. For example, disagreement over distribution of royalties in Papua New Guinea was a contributing factor in the decision of live reef fish operators to withdraw their operations from several communities [56]. Management plans that explicitly recognise and accommodate local and indigenous systems of tenure, knowledge, and resource use are more likely to be perceived as legitimate and acceptable [57], and facilitate the development of a more inclusive and equitable fishery [58,59]. Failure to respect local institutions can be detrimental; in Papua New Guinea foreign live reef fish operators were sued by communities for trespassing onto marine spaces held under customary tenure [56].

Incorporating people in planning and management processes has been shown to be a key factor for successful marine ecosystem management [60–62]. Nonetheless, small-scale fishers, who encompass migrants, indigenous groups or other marginalised people, often lack representation in decision-making processes [63]. In South Africa, for instance, artisanal and small-scale fishers experienced negative social and economic consequences when they were not recognised in the country’s post democracy fishing rights allocation system, which instead worked in favour of established fishing companies. Ultimately, small-scale fishers only gained recognition for their socio-economic rights and livelihoods following litigation [54]. The de facto privatisation of fisheries through the creation of marine protected areas and other ‘ocean grabbing’ techniques are further illustrations of violations of the collective rights of fishing communities [64].

Gender equity is a universal issue that is gaining traction in fisheries. Recognising and promoting equal rights of women in fisheries is instrumental to developing an inclusive and equitable fisheries model [65]. Women contribute significantly to socio-economic well-being at the household and wider economy [66], where most visibly they often either fish themselves to feed the family, access fish through their social network, or buy fish to supplement the family’s nutritional needs [65]. Nonetheless, women tend to earn less [67] or are relegated to minor roles in fisheries, without due consideration given to integrating them into planning and decision-making processes, or providing access to profitable fishing sectors [68–70]. Such an attitude compromises overall fisheries and social well-being, as in some cases women are better champions for transparency, conflict management, and inclusive participation in fisheries [65].

### Improve food and livelihood security in fisheries

The objective of this principle is to ensure that seafood supply chains do not operate in ways that disrupt social structures or threaten people’s ability to meet their sustenance and livelihood requirements, for example, through the imposition of low prices and economies of scale. By upholding people’s right to food and right to livelihood, this principle also captures the aims of Sustainable Development Goals to end poverty and hunger in the world [14]. Overall, it is concerned with creating conditions that improve socio-economic stability and security in communities, including protecting tenure systems and access rights to resources and markets that can increase economic opportunities.

Fisheries development that does not consider existing dependence on the targeted fish stock can lead to socio-economic disruptions, especially in food and livelihood security. The development of new fisheries can force low income fishers into heavy debt as they borrow money to acquire new capital (e.g. gear, boat) to gain entry into the new fishery. Food security may be jeopardised if the targeted fish is one that local communities have relied on for food [71], and it can also inflict changes to household nutrition and buying patterns [72]. In small-scale or subsistence-based fishing communities, women may see their provisioning responsibilities increase disproportionately to offset the financial debt. For example, women may have to spend more time fishing or gleaning for food, or take on part-time jobs that detract from their usual tasks. Gaining market access may entail building infrastructure such as new roads that open up once isolated communities to external influences, which can impact positively or negatively on the social structure and marine resource base [73,74]. Finally, while a new fishery may generate initial high financial returns, poor governance may contribute to grievances and overexploitation that can potentially threaten long-term operational sustainability [75].

### The relevance of international human rights instruments in promoting socially responsible seafood

Having identified the most prominent examples of human rights impacts within the three core pillars of socially responsible seafood, we now move to consider the ability of the current legal framework to tackle these practices. As a preliminary point, however, it must be observed that the range of international instruments and commitments that could be potentially relevant to these objectives is vast, encompassing both binding and non-binding norms. An exhaustive accounting of the legal framework that is either directly or tangentially pertinent to the promotion of socially responsible seafood is beyond the scope of this paper. For the purposes of this paper we have focused primarily on human rights instruments as the provisions most directly applicable to these issues, but other areas of law, including criminal law, labour law, maritime law and elements of environmental law will also be relevant to a greater or lesser degree depending upon context. In addition to legally binding norms, non-binding instruments, policy documents and resolutions of international and regional bodies should also not be overlooked as part of this framework. Indeed, despite their status as ‘soft’ law, non-binding instruments often act as a valuable spur for states and other actors, such as Regional Fisheries Management Organizations, to develop actions that may ultimately crystallize into standard practices and eventually provide the requisite political will for the elaboration of binding legislation [76].

Not all elements of the criteria for socially responsible seafood carry equal weight within the framework for the protection of human rights. Universal human rights are primarily articulated in two leading international legally binding instruments, the International Covenant on Civil and Political Rights (ICCPR) and the International Covenant on Economic, Social and Cultural Rights (ICESCR), which lay out the different nature and implementation of these rights. We observe a marked distinction between civil and political (CP) rights and economic, social and cultural (ESC) rights of individuals, as well as of collective rights (e.g. of indigenous people, of developing countries). ESC rights may have a lesser profile than CP rights in legal proceedings [77,78] because for example, slavery aboard international fishing vessels or child labour violations are specifically dealt with by international conventions and many have been written into national laws making them easier to enforce.

In principle, slavery remains among the most heavily restricted forms of human behaviour in both international and domestic frameworks. The absolute prohibition of slavery is among the highest forms of obligation in modern international law. The prohibition of slavery and forced labour is also recognised as a peremptory norm in international law–an obligation that is universal in character and thus binding on all states, and from which no state is entitled to derogate and any contrary legal instruments are considered null and void [79,80]. Accordingly, strict provisions against slavery are explicit in all major global and regional human rights instruments adopted in the UN era. Significantly, as exemplified by Article 4 of the European Convention on Human Rights 1950 (ECHR), the universal character of this right means that, unlike other entitlements granted to individuals, a state may not attempt to develop exceptions to this position. The Convention to Suppress the Slave Trade and Slavery 1926 defines slavery as “the status or condition of a person over whom any or all of the powers attaching to the right of ownership are exercised”, a formulation that continues to guide the modern interpretation of slavery today. Nevertheless, as observed above, there are numerous documented cases in which fishers have been maintained in conditions wherein an effective right of ownership has been exercised over their labour, hence the central problem is not a lack of legal provisions against slavery, but in their enforcement in this particular context.

Allied to slavery, the international legal framework against human trafficking and associated practices has grown significantly in recent years. Human trafficking is not a new phenomenon, and a series of international treaties were adopted during the early twentieth century purporting to combat the trade in indentured labour and enforced prostitution. The procurement of a workforce through irregular migration is currently addressed most centrally under the UN Convention on Transnational Organized Crime 2000 (the Palermo Convention) and its optional protocols on trafficking and smuggling. Efforts to broaden the concept of slavery to include exploitative practices such as forced labour, trafficking and elements of smuggling [81] has been interpreted by some courts, notably the European Court of Human Rights as having established a positive obligation to address trafficking as part of national anti-slavery obligations, thus providing an impetus to address trafficking and associated offences that have not traditionally been considered within the rather narrow ambit of slavery as articulated in older instruments. Nevertheless, at-sea enforcement operations to assist victims of trafficking remains a legal grey area [82].

In 2003, three supplementary protocols (the Palermo Protocol) to the Palermo Convention came into force. The Palermo Protocol is the basis for the UN Guiding Principles on Business and Human Rights, which instruct companies to utilize the International Bill of Human Rights to respect economic rights and to guide their human rights due diligence overall. Endorsed unanimously by the UN Human Rights Council in 2011, the principles provide a ‘Protect, Respect, and Remedy’ framework for states and companies to follow to identify risks and salient human rights issues in supply chains. These commitments have also inspired significant regional legislation to address the threats of human trafficking and forced labour in supply chains, as exemplified by the European Union and the Council of Europe. Forced labour is defined in ILO Convention 29 as “any work or service exacted from any person under threat of any penalty and for which the said person has not offered himself voluntarily.” Particular obligations requiring the prohibition and criminalization of forced labour are further established in a series of influential international instruments, including ILO Conventions 29 and 105, the Protocol to the Forced Labour Convention, and the Declaration of Fundamental Principles and Rights at Work. Smuggling, trafficking and forced labour have also been given central attention by the UN Office on Drugs and Crime in its treatment of fisheries crime [83].

The enforcement of safe and healthy working conditions within the fishing industry also raise legal and institutional complications. Traditionally, seafarers’ rights have been relatively limited and labour rights at sea have developed at a markedly slower pace than those within land-based industries [84]. The International Maritime Organization, the UN Agency responsible for marine affairs, only expressly recognised the “human element” of its remit in 1997 and has limited provision within its mandate or specialised working groups to expressly address these issues in the context of the fishing industry, considering exploitative practices almost exclusively through the lens of maritime safety. The ILO has developed a more extensive suite of seafarer protections, culminating in the adoption of the Maritime Labour Convention (MLC) in 2006. While the MLC, which entered into force in January 2013, prohibits child labour and reinforces freedom from slavery within the seafaring profession, this instrument explicitly does not apply to the fishing industry. Instead, in 2007 the ILO adopted the Work in Fishing Convention (No. 188) which entered into force in November 2017 and strives “to ensure that fishers have decent conditions of work on board fishing vessels with regard to minimum requirements for work on board; conditions of service; accommodation and food: occupational safety and health protection; and medical care and social security” (Preamble). Despite these objectives, it took nine years to acquire the ten ratifications necessary to enter into force, and no state has as acceded since–while the first and to date only detention of a vessel under this Convention occurred in July 2018 [85].

Due to their contextual and aspirational nature, ESC rights have long been treated as ‘secondary rights’ by states [78]. The weaknesses of ESC rights lie in their ambiguity in defining the nature of obligations, such as what a violation consists of, how responsibility is determined, and how to remedy a violation. These shortfalls impede effective implementation, which is further hampered by weak mechanisms to monitor and enforce compliance. These deficiencies are exacerbated by the frequent failure to recognise ESC infringements as such in the first place, which can be particularly harmful for small-scale fishers. Small-scale fisheries are prominent in developing countries, and are often exposed to governance and socio-economic conditions that threaten people’s ESC rights such as the right to health, education, and adequate food and housing. Failure to address these underlying societal issues can lead to potential inequities and erosion of fish resources, income, food security and livelihoods [34]. While these issues do not command the concerted attention associated with serious violations of CP rights, such as slavery, such outcomes are nonetheless infringements of ESC rights that jeapordise the fulfilment of several Sustainable Development Goals by 2030. Yet, given the reluctance of governments to grant domestic recognition to ESC rights [86], it will be challenging from a legal and practical perspective to use ESC rights as a strategy to hold governments accountable for ensuring the well-being of fishing communities.

### Challenges and opportunities in applying human rights to implement socially responsible seafood

There has been a relatively limited tradition of attempting to use the current tapestry of human rights instruments to promote socially responsible practices in the seafood sector. A primary concern is that the legal reach of human rights does not always extend to cover the issues and stakeholders that are involved in ensuring social responsibility along the entire seafood supply chain. The human rights instruments developed in the decades following the founding of the UN were primarily intended to prevent the types of atrocities against humanity that were witnessed during World War II. Moreover, guiding human rights principles that could more clearly underpin commitments towards producing socially responsible seafood have emerged more recently and, as such, have not attained the same legal traction as the longstanding CP rights outlined above. For example, the right to natural resources has yet to be universally recognised as a human right. This has clear practical limitations for certain fisheries constituents in advocating community control over natural resources as a human right [87]. A universal recognition of a right to natural resources would undoubtedly strengthen individual claims towards securing people’s access to resources and thus facilitate the attainment of one of the central principles of socially responsible seafood.

In practice, socially responsible seafood is a market-driven initiative in which seafood companies bear primary duties. Human rights treaties rarely impose legal obligations on private actors, which raises the question of how to hold seafood companies accountable for failing to secure socially responsible outcomes. In many key respects, international law remains a fundamentally consensual system and the decision to participate in treaty regimes that may be of particular value to fishers’ rights remains an individual choice exercised by states based on their political and cultural sensitivities. As evidenced by the limited participation in the ILO’s Work in Fishing Convention, especially by ‘developed states’, there has been a marked reluctance to endorse treaties that directly impact upon the working conditions of fishers. Moreover, where human rights treaties have been ratified, they must be assiduously implemented by the national authorities and expressly applied to the maritime sector in order to inspire meaningful outcomes. Legally, seafarers have traditionally been treated in isolation to the terrestrial workforce as a special class of labour and a disappointingly high number of countries have failed to apply equivalent protections for vital rights such as fair and timely wages, freedom of assembly and association and safe and healthy working conditions for ship-based work [84]. Likewise, there may be loopholes within key legal provisions that may have unintended negative consequences for seafarers. Notably, ‘developing countries’ are not obliged to extend the economic rights granted under the ICESCR to non-nationals, thereby allowing particular signatories to legitimately fail to protect the interests of some of their most marginalised at-sea workers [88]. Likewise, the Work in Fishing Convention allows for a series of exceptions that “effectively exclude a significant number of fishing vessels from the scope” of the Convention [89], thereby curtailing its prospective impact significantly even if it were to be widely ratified.

Compounding these difficulties, while international instruments may establish particular entitlements, the enforcement of these principles is not straightforward. Aside from the ECHR, the right to individual petition is often an optional entitlement that many flag states have chosen not to accept [88]. The Work in Fishing Convention allows “any person with an interest in the safety of the vessel” to register a complaint about working conditions, but as observed above few states have ratified this instrument. Likewise, the International Tribunal for the Law of the Sea does not accept individual petitions and has routinely disregarded *amicus curiae* (“friend of the court” or neutral third party) interventions raising matters concerned specifically with human rights [90]. On a national level, however, domestic provisions may prove to be rather more far-sighted. In the USA, the Department of Homeland Security has the authority to initiate an investigation into forced labour or child labour in seafood or any goods based upon a petition submitted by any person. A list of goods made with a significant incidence of forced labour is available free of charge to the public in the Sweat and Toil smartphone application published by the U.S. Department of Labor.

Beyond these conceptual challenges, it is also apparent that significant practical difficulties have been encountered in seeking to apply the network of human rights treaties addressing both civil and political and economic, social and cultural rights of fishers. Among seafarers there is generally a chronic lack of awareness of the range of right to which they are entitled [84,88]. Even where fishers have this knowledge–or violations of their rights are so egregious that there are self-evident breaches of national and international laws–such persons will often lack the resources to attempt to assert their rights in any event. Moreover, there is little active support or assistance for such persons to bring a legal claim in the jurisdictions in which they are most at risk. Where advocacy groups or activists are active in such jurisdictions, they may face harassment and intimidation, including through strategic lawsuits and amorphously defined legal provisions, such as defamation, civil disobedience and the expansive concept of national insult. For example in 2018 criminal defamation cases were filed in Thailand against human rights campaigner Andy Hall, a foreign activist who had vociferously highlighted abusive conditions in the country’s supply chain [91]. As such, human rights hold promise at a conceptual level but their inherent limitations have to be recognised in practical efforts to apply the principles of socially responsible seafood.

Notwithstanding the challenges described above, there are opportunities for using human rights laws to achieve the promise of socially responsible seafood. Governments can draw upon international instruments to frame national laws that can then be applied to address human rights violations in fisheries. Recent examples include the United Kingdom, which in 2015 enacted the Modern Slavery Act [92], which strengthens law enforcement against instances of modern slavery, including those seen in global fisheries. At a late stage in the debate, a well-publicised report on labour violations within the national fishing fleet raised awareness of the hitherto little considered plight of fishers [45], leading to the addition of further powers of at-sea enforcement. In New Zealand, the government responded to a series of non-legislative measures against forced labour at sea with the Fisheries (Foreign Charter Vessels and Other Matters) Amendment Act which came into force on May 1, 2016. This Act requires any foreign fishing vessel operating in New Zealand waters to reflag as a New Zealand ship, removing their right to fish in New Zealand waters until they do so, and is intended to help identify forced labour and ensure New Zealand employment law is applied uniformly at sea. During the first reading of the Act, the government acknowledged that forced labour in the fishing industry had resisted successive government measures for at least the last two decades [93]. This demonstrates that governments can be receptive to targeted advocacy towards the need to promote socially responsible seafood.

The USA has also sought to combat illegal fishing by passing the Illegal, Unreported, and Unregulated Fishing Enforcement Act [94]. Since 2017, aspects of the legislation are supported by a risk-based traceability program, the Seafood Import Monitoring Program, which establishes reporting and recordkeeping requirements for imports of certain seafood products, to combat illegal, unreported and unregulated (IUU)-caught and/or misrepresented seafood from entering U.S. commerce. There is also U.S. federal law that prohibits the import of goods that have been produced by forced labour (Smoot Hawley Tariff Act 1930). In October 2018, an Interagency Task Force on Forced Labor in International Waters was established by the Department of Justice due to federal “concerns of labor that may have been subject to human trafficking to harvest fish in international waters”, which could lead to additional rules for seafood companies for social accountability. While these advances are to be celebrated, the violations that national laws in market countries address tend to be associated with the civil rights of individuals who often are foreign workers. While enacting relevant laws can be effective at national or sub-national levels in forcing seafood companies to seriously look at accountability and transparency in their supply chain [95], to ensure that human rights are respected at every stage, there has to be cumulative buy-in and motivation across institutional levels to enforce them consistently.

Where legal regimes have been ineffective, non-binding ‘soft’ laws can fill the gap left by ‘hard’ binding legislation. Although non-binding in nature, soft laws can contain elements that foster compliance, and are especially suited to address issues that lack consensus or for which governments are wary of making legally binding commitments [96]. They thus represent an intriguing avenue to promote objectives that have not gained universal and binding support. The FAO’s Voluntary Guidelines on the Right to Food in 2004 was a soft law approach to advance the right to food. It has had positive impact on several fronts, including improving implementation of the right to food at the policy level, shifting prevailing perceptions around the impracticality of ESC rights, and promoting discourse between international development and human rights agendas [78]. A similar approach may likewise be appropriate for progressing to socially responsible seafood.

## Discussion

Creating a definition of socially responsible seafood that encompasses the full spectrum of civil and political, and economic, social, and cultural rights is a first step in drawing attention to the distinct aspects, thus different treatment, that is required to protect fisheries workers’ rights and well-being [12]. The research landscape for socially responsible seafood showed that the focus on socio-economic and cultural rights is highly relevant to small-scale fisheries, many of which operate under poor governance and socio-economic conditions which in turn threaten the food security, health, and livelihood of fishers. However, simply relying on the human rights framework to protect fishers’ socio-economic well-being may prove to be rather a blunt instrument where national laws do not implement pathways to secure the full range of ESC rights, or where the national authorities simply disregard the human rights of particular constituencies. Here, human rights more usefully serve as universal moral and ethical standards that legitimise people’s claims against injustice. In Uganda, peasant farmers turned to a rights-based approach to seek redress after their lands were forcefully taken away by the government for foreign investment interests [87]. This illustrates that even if human rights laws are weak, evoking them nonetheless can spur events to influence future change, such as by challenging oppressive behaviour or rallying public support for corrective action. It is also worth noting that human rights can be elaborated to better accommodate the needs of oppressed groups as they become apparent, as was the case with the UN Declaration on the Rights of Indigenous Peoples which was created specifically to deal with discrimination faced by indigenous peoples globally.

Several governments have responded to criticisms of human rights abuses in fisheries by enacting regulatory changes and improving national policies [97]. These changes have largely related to forced labour, health and safety, and other labour rights–civil and political rights–where clear distinctions can be made to determine criminality, i.e., whether a violation has occurred. For instance, human rights abuses in the fisheries sector that have gained media exposure and created consumer awareness are dominated by slavery and human trafficking, which are clear violations of CP rights. In contrast, violations of ESC rights are more subtle and easily overlooked because they are often hard to separate from the fishing communities themselves. For example, poverty in small-scale fisheries is pervasive and is a major obstacle to such necessities as food security and education for children. Yet, these conditions are seldom considered infringements in the same way that slavery is viewed as an especially egregious human rights violation by fisheries managers and seafood consumers. That said, there are examples where customary rights to fisheries have been enshrined in national domestic laws [98]. Moreoever, in the Philippines the government has responded to social injustice concerns in the fisheries sector by moving towards decentralised natural resource management, where fishing communities are allocated certain power in co-management arrangements [99].

The rights-based approach to fisheries [7], in which fisheries development is pursued in step with developing capacities to actualise human rights, elucidates the linkage between human rights and fisheries. It has been adopted in the Sustainable Development Goals, for example in target 1.4 to ensure by 2030 that all men and women, in particular the poor and the vulnerable, have equal rights to economic resources, and in major guidance documents such as the FAO’s SSF Guidelines as a way of bringing ESC rights to the fisheries sector. This provides a useful foundation for making progress in recognising and addressing the lack of ESC rights in fisheries, where the biggest challenge is likely summoning sufficient political will to create or adequately support the legal structures needed to implement these principles effectively [100]. Encouragingly, there are positive signs that national policy arenas have been receptive towards the SSF Guidelines [101,102], and if advanced further, the SSF Guidelines could become an instrument through which national governments may institutionalise ESC rights.

The path to meeting ESC rights in the seafood supply chain is not without challenges, not least because of the financial commitments or political reforms which will likely be involved. In different parts of the world changes that are needed to meet the desired economic, social and cultural mandates of socially responsible seafood might mean better access to health and education services, demarcation of fishing grounds, or formation of co-operatives [60]. A useful way of grounding the vague language of ESC rights is to think of what it would take to incorporate the most marginalised fisher community into the seafood supply chain. Often, this will reveal gaps where there are shortfalls in meeting economic, social, or cultural needs. Difficult questions such as whether fish caught by politically or socially marginalised people should be considered socially responsible will have to be addressed. Are seafood companies prepared to not only respect but also champion fishers’ rights? Without meeting basic livelihood necessities, it will be difficult to engage fisheries workers to think beyond their immediate needs and embrace marine resource conservation and sustainable fisheries management. Yet the types of structural changes that are required for transitioning to socially responsible fisheries will lead to broader overall governance improvements and progress towards the global and rights-based Sustainable Development Goals [14], especially in developing countries.

Drawing on market forces may be another alternative where merely appealing to ESC rights laws has proved incapable of delivering the societal improvements sought by socially responsible seafood. Although responsibility for protecting human rights falls on national governments, this does not absolve companies from performing due diligence to ensure respect for human rights. According to the first pillar of the UN’s ‘Protect, Respect and Remedy’ framework [103], governments have a duty to guard against human rights abuses by third parties, including business enterprises. There is growing consensus that corporations should at a minimum respect human rights at all times during in their operations, or to go even go further and actively prevent potential human rights violations from arising as a result of their operations [104]. Tools are increasingly becoming available for this purpose. For example, the Seafood Slavery Risk Tool helps businesses to identify the potential risk of slavery, human trafficking, or child labour in their fisheries supply chain, while companies can use the Labor Safe Screen framework for conducting human rights due diligence [13]. With a critical mass of corporations adopting the ‘Protect, Respect and Remedy’ framework, market pressure from investors and consumers can become effective drivers of good corporate behaviour. The motivation for securing human rights then shifts from being one of merely avoiding liability, to one of actively promoting socially responsible behaviour. For instance, companies can create pricing and profit-sharing schemes that generate higher incomes for fishers, thus fulfilling the socially responsible principles of equity and equality, as well ensuring as food and livelihood security.

However, relying on market solutions has to be undertaken with caution, with an eye towards ensuring there is alignment in interests between supply chain actors and socially responsible seafood principles. For instance, it has been demonstrated that seafood attributes that are important to traders and consumers in Asia pertain to food safety, product quality, and cultural status [105]. Subsequently, supply chain components will be modified to deliver on those requirements rather than the social issues that concern fishers. Other sectors with longer engagement in social sustainability have seen uneven results in social benefit outcomes. More powerful producer organisations have tended to gain more from participating in Fair Trade coffee compared to marginalised producers, while Forest Stewardship Council certification has mainly benefitted northern countries and operations that are already well-managed [106]. Within fisheries, sustainable aquaculture certification schemes in Vietnam were considered more likely to benefit large companies rather than small-scale producers [107], and the suitability of MSC certification for developing countries has been debated [11,108]. Ensuring that the focus on fisher well-being is not lost is all the more important considering the recent elevation of ‘keystone actors’ [109]–in which large international fishing corporations are entrusted with transitioning the seafood sector towards sustainability–as a platform for ocean stewardship.

## Conclusion

We showed that the seafood supply chain is currently operating in a landscape fraught with social abuses that impinge upon the full spectrum of the civil and political rights, and economic, social and cultural rights of individuals and groups. These violations have persisted in part because of insufficient pressure on and motivation from the seafood industry and governments to tackle the problem. This arises due to a failure to define rights to fish as essential to local people in state legal frameworks, and to corruption, but also due to the absence of a coherent vision of the desired outcome, and ambiguity or a lack of knowledge about the nature of the issues at hand and the process and tools that can be used to achieve the desired outcome. Aspirations for socially responsible seafood provide a clear objective for the seafood sector in which social abuses are to be at least reduced and preferably eliminated. This goal demands that the fundamental human rights of all individuals should be upheld; realising human rights for fishery workers is thus an obligation and forms the premise for pursuing socially responsible seafood. Nonetheless, there are limitations in the extent to which the human rights framework can be harnessed in order to realise this objective.

There is no shortage of international human rights instruments that support the guiding principles of socially responsible seafood. However, human rights enshrined in international treaties may not always address the full scope of issues to ensure that seafood is considered socially responsible under varying local contexts, and will likely need to be complemented by improved methods of recognizing bottom-up approaches and market-based alternatives. Soft laws or voluntary standards can effectively fill in the gaps left by the weak application of human rights laws. With sufficient endorsement by stakeholders along the seafood supply chain, voluntary standards can become the industry operating norm and thus achieve further legitimacy and compliance. For fisheries workers, human rights serve to legitimise their claims and instigate corrective action against social abuses where they occurs, even if the human right framework is in and of itself imperfect. Market reforms alone are insufficient in the context of small-scale fisheries to achieve the principles of socially responsible seafood. Rather, protecting economic, social and cultural rights will require broader institutional changes in local governance and social development, such as opportunities for legal pluralism like co-management, a perspective that is shared in the FAO’s SSF Guidelines. In conclusion, implementing socially responsible seafood on the ground will require actions on multiple fronts, where desired social outcomes are traced to target a broader set of rights and parties that have the capacity to influence those rights.

## Supporting information

S1 File Literature search strings. (DOCX)
