# Supplementary material for: The role of human rights in implementing socially responsible seafood
Source: PLoS One. 2019 Jan 25;14(1):e0210241. doi: 10.1371/journal.pone.0210241 (PMC6347265; doi:10.1371/journal.pone.0210241)
Supplement: S1 File — (DOCX) [file pone.0210241.s001.docx]

**S1 Appendix: Literature search strings**

1. Terms used in first search:

“human rights” + “fisheries”

“human rights” + “fishing”

“human rights” + “fishing” + “crew”

“human rights” + “fish workers”

“human rights abuses” + “fisheries”

“human rights abuses” + “fishing”

“human rights abuses” + “fishing” + “crew”

“human rights abuses” + “fish workers”

“human rights violations” + “fisheries”

“human rights violations” + “fishing”

“human rights violations” + “fishing” + “crew”

“human rights violations” + “fish workers”

2. Terms used in second search:

“fisheries” + “child labor”

“fisheries” + “human trafficking”

“fisheries” + “forced labour”

“fisheries” + “slavery”

“fisheries” + “migrant fishers”

“fisheries” + “illegal workers”

“fisheries” + “labor standards”

“fishing” + “child labor”

“fishing” + “human trafficking”

“fishing” + “forced labour”

“fishing” + “slavery”

“fishing” + “migrant fishers”

“fishing” + “illegal workers”

“fishing” + “labor standards”

“fish processing” + “child labor”

“fish processing” + “human trafficking”

“fish processing” + “forced labour”

“fish processing” + “slavery”

“fish processing” + “migrant fishers”

“fish processing” + “illegal workers”

“fish processing” + “labor standards”

3. Search strings that did not produce additional case studies:

“social rights violations” + “fisheries”

“social rights violations” + “fishing communities”

“social rights violations” + “fishers”

“indigenous rights” + “fisheries”

“indigenous rights” + “fishing communities”

“indigenous rights” + “fishers”

“cultural rights” + “fisheries”

“cultural rights” + “fishing communities”

“cultural rights”+ “fishers”
